# Seasonal *Bordetella pertussis* pattern in the period from 2008 to 2018 in Germany

**DOI:** 10.1186/s12879-020-05199-w

**Published:** 2020-07-03

**Authors:** Daniel Allermann Hitz, Friedemann Tewald, Maren Eggers

**Affiliations:** Laboratory Prof. Gisela Enders and colleagues, MVZ, Stuttgart, Germany

**Keywords:** Pertussis, Whooping cough, Seasonality, Germany

## Abstract

**Background:**

After the introduction of a vaccine against *B. pertussis* the seasonal pattern with the highest number of infections in the spring to summer months changed. Recent studies from around the world suggest that *B. pertussis* infections again follow a seasonal pattern with increased incidence in summer.The aim of this study was to investigate whether respiratory infections caused by *B. pertussis* in the period from January 2008 to December 2018 also seasonally spread in Germany and if so, when the *B. pertussis* activity peaked.

**Methods:**

We tested 19,031 samples, mainly from Southern Germany, collected in the period from January 2008 to December 2018 using a Multiplex PCR assay. We assessed the number and proportion of samples positive for *B. pertussis*, stratified by patient’s age and month. The seasonal distribution was investigated by plotting the average proportion of positive samples for each month.

**Results:**

We observed a *B. pertussis* seasonality with the highest number of positive samples in the months from June until September. In contrast, testing of samples for *B. pertussis* was requested most frequently in the period from October until March. The proportion of positive samples increased earlier in adolescents (age 10 to 19) than in other age groups.

**Conclusions:**

We found a seasonality of *B. pertussis* infections in Germany, which differs from the time when most samples are sent in for testing of *B. pertussis.* Our study suggests that clinicians should be more aware of *B. pertussis* infections in the months from June until September to prevent further transmission to vulnerable family members.

## Background

Whooping cough, also known as pertussis, is a respiratory disease that is predominantly caused by the gram-negative bacterium *Bordetella pertussis*, which is found only in humans [1]. For older children and adults the disease is mostly mild, but infants younger than 6 month of age risk severe complications and even deadly infections [2, 3]. Rarely, whooping cough can also be caused by the gram-negative bacteria *Bordetella parapertusis, Bordetella holmesii*, and *Bordetella bronciseptica* [4, 5]*.*

In the pre-vaccine era whooping cough followed a seasonal pattern with the highest number of cases in the spring [6] and/or summer months [7], however with the introduction of a vaccine against *B. pertussis* this pattern was less pronounced [6] or even absent [8].

In recent decades, the incidence of *B. pertussis* infections increased in countries with high vaccine coverage, which could have been caused by 1) genetic changes 2) decreased vaccine uptake or vaccine efficacy 3) waning immunity after immunization 4) better surveillance 5) new sensitive diagnostic tests or 6) age-structured contacts [9, 10]. In Germany, the incidence of whooping cough increased from 16.8% in 2016 to 20.5% in 2017 [11, 12].

Recently, several studies from around the world suggest that *B. pertussis* infections again follow a pronounced seasonal pattern. An American study found that the proportion of *B. pertussis* positive samples was lowest in the months of October and February and highest in July and August. It peaked late in July, where it was five times higher than in late February [13]. In Europe, the highest number of cases was reported in June and September and the lowest number in January and February to the European Centre for Disease and Control (ECDC) in 2016 [14]. In China, a study found that the number of reported cases was highest in the period from June until September [15]. Additionally, an Australian study found that most cases of whooping cough occurred in their summer months - between October and February (southern hemisphere), while the highest number of tests were performed in their winter and spring months [16]. However, a Danish study found that whooping cough had a less pronounced seasonal pattern with a higher number of infections in the months from August to November and a lower number between February and April [17].

The aim of this study was to investigate whether respiratory infections caused by *B. pertussis* have also shown a seasonal distribution in Germany in the period from January 2008 to December 2018 and if so, when the *B. pertussis* activity peaked. In addition, it was to be assessed whether the number of samples sent to the laboratory for *B. pertussis* tests coincided with the seasonality identified.

## Methods

### Design

The anonymised data in this retrospective study are gathered from routine *B. pertussis* testing in our laboratory from January 2008 to December 2018.

### Samples

Material was sent to our laboratory for routine *B. pertussis* analysis. The origin of the study samples were in 7% hospitals and in 89% private practices of pediatricians and general practitioners in Southern Germany. In 4% the origin of samples was not documented.

The clinical specimens comprised of 80% nasopharyngal swabs (cat. No. 160c rayon mini tip, Copan 100 Diagnostics Inc., USA) and 20% less suitable materials like sputum, gargle samples and gels swabs. Clinical specimens were delivered to the laboratory within one or 2 days after collection in 1 ml NaCl. Prior to DNA extraction, bacteria were collected by centrifugation in a 1,5 ml microfuge tube at 13000x g for 5 min. Pellets were resuspended in 200 μl H_2_O and processed as described below.

### Automated nucleic acid isolation

DNA was prepared with the automated MagNA Pure instrument (Roche Diagnostics GmbH, Germany) by using the MagNA Pure LC total nucleic acid isolation kit (Roche Diagnostics GmbH, Germany), the QIAcube™ (QIAGEN, Venlo, Netherlands) or the NUCLISENS® easyMAG® (BioMérieux, Germany). Briefly, the input sample volume was 200 μL and nucleic acids were eluted in a volume of 50 μL. Purified DNA was stored at − 20 °C. Genomic DNA of mouse cells used as internal control for monitoring DNA extraction and PCR inhibition was co-extracted with each sample [18].

### PCR analysis

A real-time multiplex PCR assay targeting the insertion sequence elements IS481, IS1002 [19], and the insertion sequence IS1001 [20], were used to detect *Bordetella* spp. in respiratory specimens.

In addition, to improve the specificity of the IS 481-based PCR assay, the hIS1001 target was included in 2016 in order to detect *B. holmesii* [21]. Table 1 shows how the results from the real-time PCR multiplex assay were interpreted.

**Table 1:** Identification of Bordetella species

Results of the real-time multiplex PCR assay				Interpretation
IS481	IS1001	hIS1001	IS1002	
Positive	Negative	Negative	Positive	*B.pertussis*
Negative	Positive	Negative	Positive	*B.parapertussis*
Positive	Negative	Positive	Negative	*B.holmesii*

Identification of the *Bordetella* species are based on results from the real-time multiplex PCR assay testing four different target sites

TaqMan probes for *B. pertussis, B. parapertussis, B. holmesii, Bordetella* spp. and internal control were labelled with reporter dyes FAM™, JOE™, Cy5.5™, ROX™, and Cy5™ respectively. Master mixes were based on the LightCycler FastStart DNA Master HybProbe kit (Roche Applied Science, Germany). Each 20 μl reaction contained the following components: 4.5 mM MgCl_2_, primers of IS481, IS1001 and hIS1001 at a concentration of 0.5 μM each, primers of IS1002 at a concentration of 0.75 μM each, primers for IC at a concentration of 0,06 μM each, 2 μl of FastStart DNA Master HybProbe (10x con.), 0.06 μM of each TaqMan probe, and Uracil-DNA Glycosylase (0.5 U). Amplification conditions on the Rotor-Gene 6000 (Qiagen, Germany) consisted of three consecutive phases: (i) an initial denaturation step in order to activate the FastStart Taq DNA Polymerase (95 °C for 10 min), (ii) a touch-down profile for 10 cycles (95 °C for 1 s, 68 °C for 10 s) lowering the annealing temperature by 1 °C every cycle, (iii) an amplification step for 40 cycles (95 °C for 3 s, and 58 °C for 10 s). Data were analyzed with the Rotor-Gene software 2.1.0 (Qiagen, Germany). For all real-time PCR assays, cycle threshold (Ct) values < 35 were considered positive. A non-template PCR negative control (sterile nuclease-free water) and positive template control were included in each run.

### Statistical methods

To study the seasonal distribution of *B. pertussis*, the average number, the average proportion, and the median proportion of positive samples for each age group were calculated and plotted on the vertical axis and months on the horizontal axis. All calculations and visualizations were performed using Microsoft Excel 2010 or Rstudio [22]. Comparison of proportions was performed by using the prop.test function in Rstudio. Calculated as 95% exact Clopper-Pearson confidence intervals.

### Ethics statement

Ethical approval was not required for this retrospective study since all samples were sent to our laboratory for routine laboratory *B. pertussis* analysis and they were anonymised before statistical evaluation. The study was carried out in compliance with the Helsinki Declaration.

## Results

In the period from January 2008 to December 2018, the laboratory received a total of 19,031 samples from 17,962 patients for *B. pertussis* testing. 15% (*n* = 2918) of the samples were *B. pertussis* positive. Median age of all patients was 6 years (IQR: 1–15) and median age for patients with positive samples was 8 years (IQR: 3–12).

**Fig. 1:**
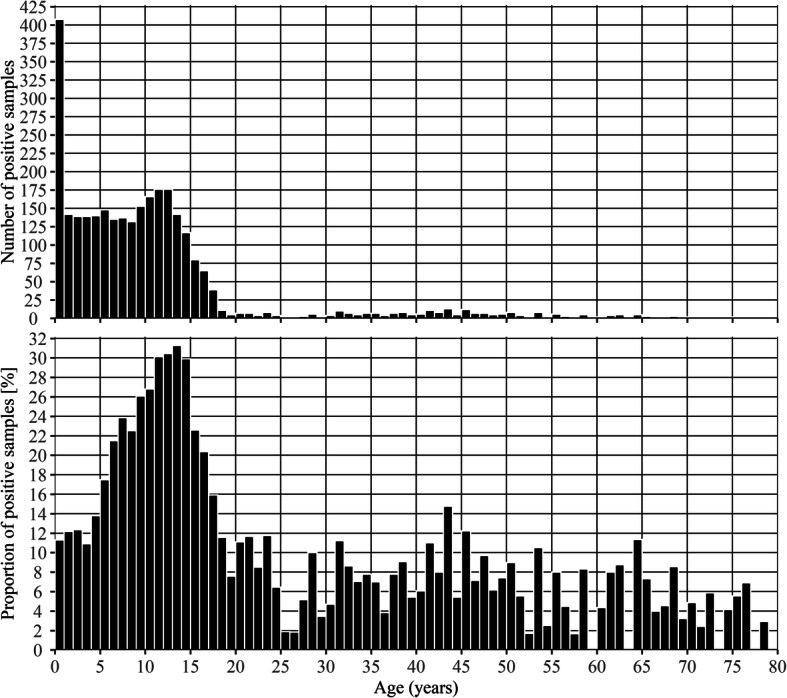
Number and proportion of positive samples by age in years. The upper part (**a**) shows the number of positive samples and the lower part (**b**) shows the proportion of positive samples by age in years

The highest number of positive samples (*n* = 408) was observed among patients younger than 1 year of age, but the highest proportions of positive samples were observed in patients between 5 and 17 years of age with a peak (31%) around the 13th year of age (Fig. 1).

**Fig. 2:**
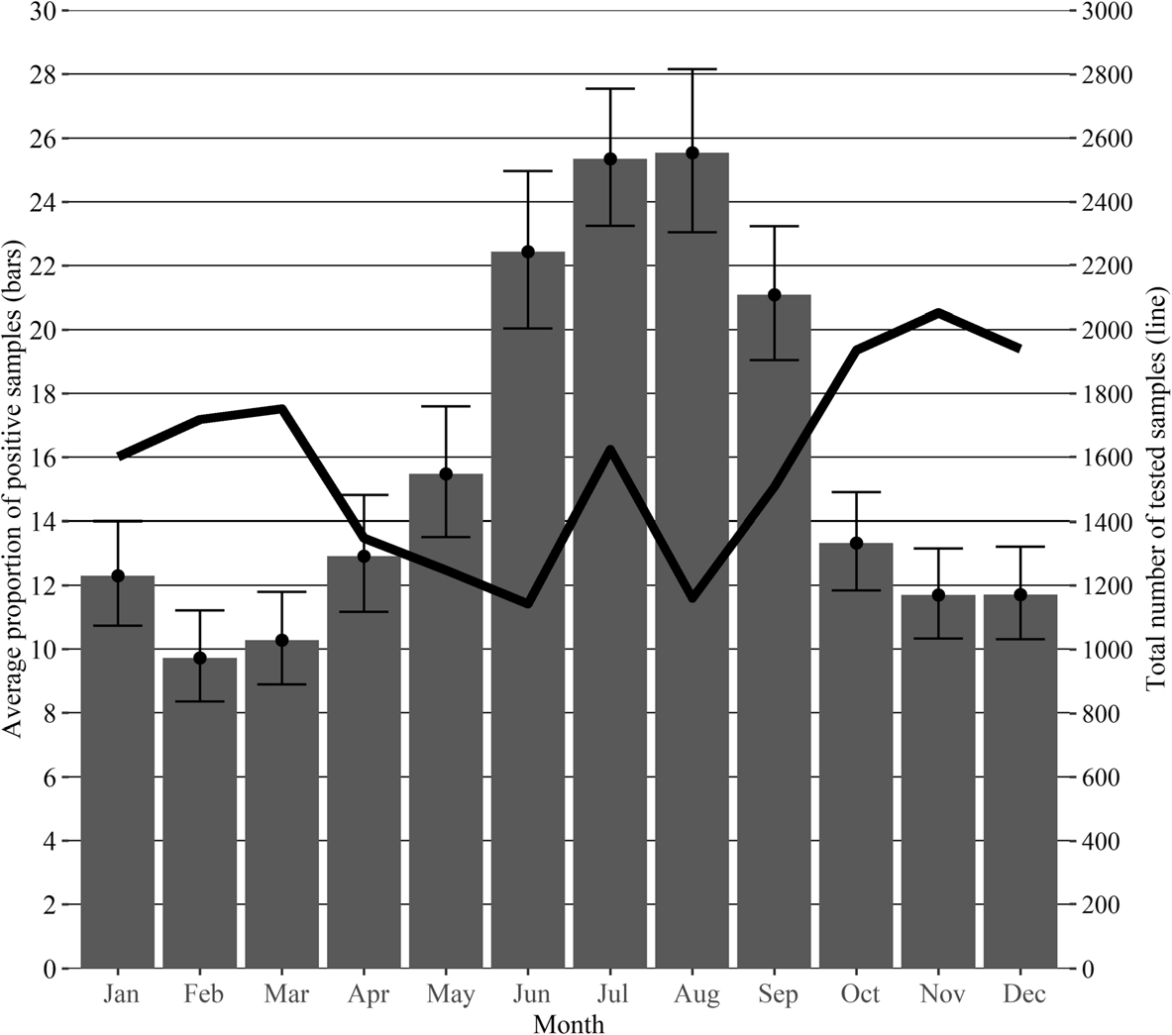
Average seasonal pattern of positive samples during 2008–2018. The bars show the average proportion of positive samples with 95% confidence intervals for January to December in the study period. The black line shows the average total number of tested samples for January to December in the study period

In the study period, the following average seasonal pattern has emerged: the highest proportions of positive samples were found in the period from June until September, peaking in July and August, and the lowest were found in the months from October until April (*p* < 0.01) (Fig. 2). The average proportion of positive samples was more than two times higher in July and August (26%) than in February (10%) (Fig. 2). However, as shown by the black line in Fig. 2 the total sample numbers sent to the laboratory for whooping cough diagnosis were highest (max. 2053) in the period from October until March and lowest (min. 1141) between April and August, except for a peak in July (1625) (Fig. 2).

**Fig. 3:**
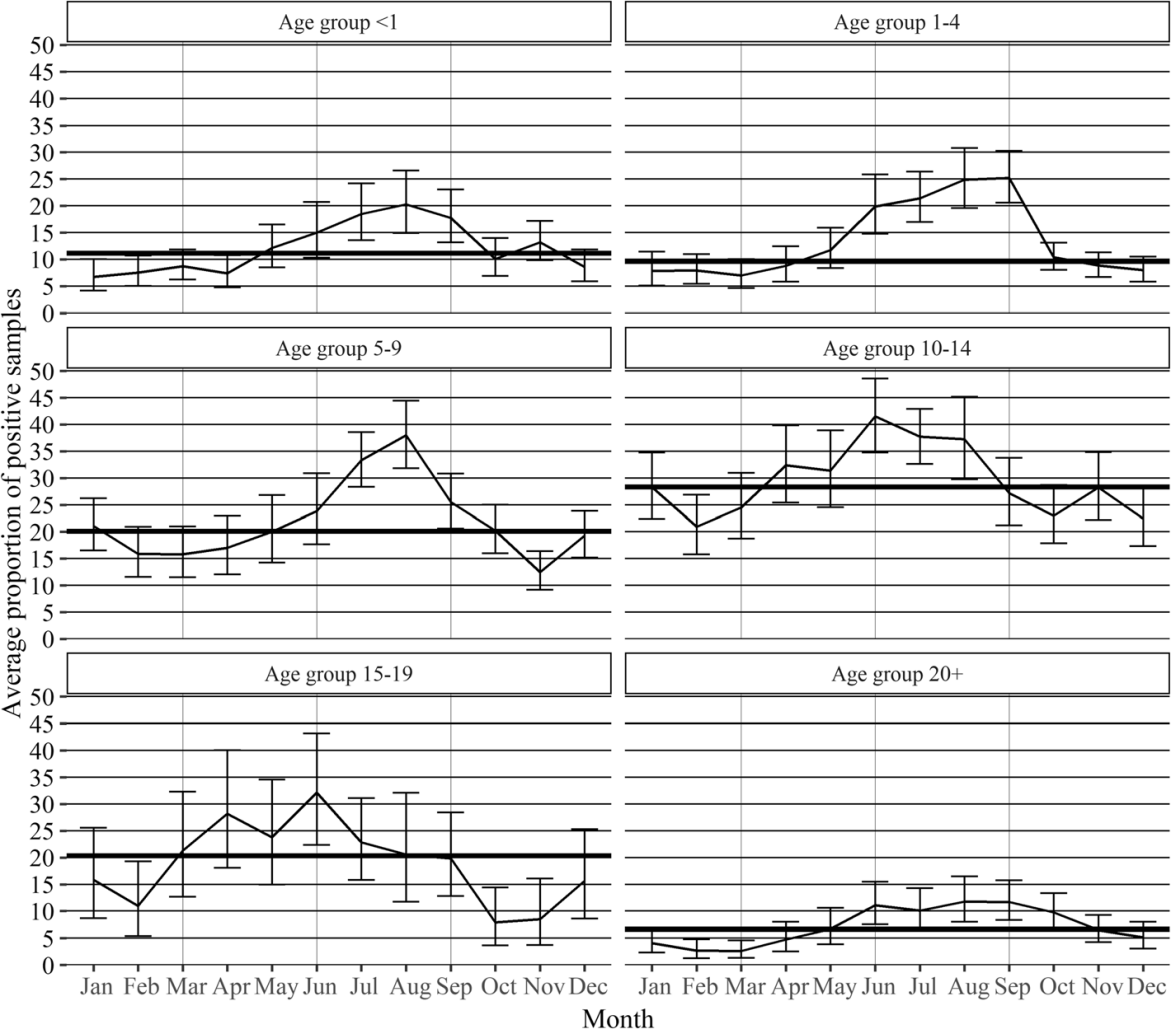
Average seasonal pattern of positive samples in each age group during 2008–2018 (proportions). The lines show the average proportion of positive samples with 95% confidence intervals in each age group for the months January to December in the study period. The bold line shows the median proportion of positive samples in each age group

As shown in Fig. 3, the average proportion of positive samples vary from month to month in all age groups (*p* < 0.05). The median proportion of positive samples were 11.1, 9.6, 20.1, 28.3, 20.3, and 6.6% in the age groups “< 1”, “1–4”, “5–9”, “10–14”, “15–19”, and “20+”, respectively (Fig. 3).

The first age group to have proportions above its median proportion of positive samples (bold line) was the age group” 15–19″ from March until August, followed by the age group “10–14″ from April until August, the age group “< 1″ from May until September, the age group “1–4″ from May until October, the age group “5–9″ from June until October, and the age group “20+” from May until October (Fig. 3).

## Discussion

Our findings showed that *B. pertussis* infections indeed can be observed throughout the year, but the proportion of positive samples increased in the summer months peaking in July and August. However, the number of samples sent in for whooping cough testing in our laboratory followed a different pattern, as the number of samples was higher in the autumn and winter months than in the summer months (excluding July).

As a matter of fact, the number of positive samples can be influenced by the number of samples tested, which again can be influenced by the epidemiological situation. However, our results showed a high number of positive samples in months with a low number of tested samples (data not shown), so we do not suspect the epidemiologic situation to have any relevant influence on the interpretation of our results.

Additionally, our findings support the recently published studies from the USA, Europe, Australia, and Asia, which all found a higher proportion of positive samples in the summer period than in the winter period [13, 15, 16, 23, 24]. Bhatti et al. showed a 5-fold increase in the proportion of positive samples in the USA, which is even larger than the more than 2-fold rise found in our study [13]. In 2016 the highest number of cases in Europe were reported to the ECDC in June and September and the lowest number in January and February [14]. Also, an Australian study reported a higher proportion of positive samples in their summer months (southern hemisphere) between October and February (mean = 13.5%) than between March and September (mean = 6.9%) [16].

Other studies also reported that the number of requested laboratory tests did not follow the same seasonal pattern as whooping cough [13, 16, 24]. Bhatti et al. observed a 2-fold rise in the number of samples tested for *B. pertussis* in November (*n* = 857) compared to July (*n* = 430) in the USA, which is even larger than the rise observed in our study [13].

According to the guideline on whooping cough from the Public Health Institute for Germany (Robert Koch Institute) it is shown, that whooping cough is found all throughout the year with slightly more infections in fall and winter than in the rest of the year [1]. However, 35% of whooping cough cases reported to the Robert Koch Institute are not included in their statistics since not all of the required information according to the case definition are at hand, which may underestimate the incidence of whooping cough in their statistics [25, 26]. This corresponds to the results of an Australian study demonstrating that the number of notifications for whooping cough followed the overall trend for *B. pertussis* testing and that the seasonality of whooping cough was less clear, when statistics for whooping cough infections were based on notifications [16].

These discrepancies may be an indication that patients with symptoms of whooping cough could be interpreted differently, if presented to the clinician at another time in the year other than the fall and winter period [13]. In addition, Bhatti et al. indicate, that the true incidence of whooping cough may be underestimated due to the unawareness of the seasonality of whooping cough amongst the clinicians [13].

Additionally, the age groups “15 – 19” and “10 – 14” got infected earlier in the year compared to the other age groups in this study. This is in contrast to King et al. and de Cellés et al., who reported late autumn seasonal peaks for this age group in Massachusetts [27, 28]. On the other hand, a Dutch study described a peak in the age group “13–18” in summer similar to our study and one in autumn such as in the American studies [23]. In our study, the increase of infections in the age groups “15–19” and “10–14” begins in March and April instead of May as in the age group of < 5 years old children. This may indicate differences in social interaction between the different age groups [29], but could also be an indication of different periods of increased transmission in the age groups [23]. Greeff et al. hypothesized that this difference in time of infection might be explained by the fact that children attending school might get infected after an exhaustative school year and then afterwards infect their family in the summer break [23]. Nevertheless, our study supports the suggestion by King et al. that teenagers are a core transmission group [27]. Moreover, the results of de Cellès et al. show that children and adolescents in particular may play a more important role in the transmission of whooping cough than adults, perhaps even a key role due to the frequency of contacts and social interactions in the respective age groups [28].

## Conclusion

Our results showed a clear seasonal pattern with the highest proportions of positive samples in Germany during the summer months from January 2008 to December 2018. In adolescents (age 10 to 19) the proportion of positive samples increased earlier than in other age groups. Our study suggests that clinicians should be more aware of *B. pertussis* infections in the summer months, especially in vaccinated adolescents and adults with less severe symptoms, to prevent further transmission to vulnerable family members.

## Data Availability

The datasets used and/or analysed during the current study are available from the corresponding author on reasonable request.

## Abbreviations

ECDC: European Centre for Disease Control
DNA: Deoxyribonucleic acid
PCR: Polymerase chain reaction

## References

[1] Robert Koch-Institut. Keuchhusten (Pertussis); 2017 [Cited 2019 Dec 2]. Available from: URL: https://www.rki.de/DE/Content/Infekt/EpidBull/Merkblaetter/Ratgeber_Pertussis.html.

[2] Riffelmann M, Littmann M, Hülße C, Hellenbrand W, Wirsing von König CH (2008). Pertussis: not only a disease of childhood. Dtsch Arztebl Int.

[3] European Centre for Disease Prevention and Control. Expert consultation on pertussis. Stockholm: ECDC; 2012 Nov 20 [cited 2019 Dec 17]. Available from: URL: https://www.ecdc.europa.eu/sites/default/files/media/en/publications/Publications/pertussis-meeting-2012.pdf.

[4] Gross R, Keidel K, Schmitt K (2010). Resemblance and divergence: the “new” members of the genus Bordetella. Med Microbiol Immunol.

[5] Schimmel D, Hänel I, Hotzel H, Hillert R (1998). Infektionen des Menschen mit Bordetella bronchiseptica und Infektionen beim Pferd. Bundesgesundhbl..

[6] Gomes MC, Gomes JJ, Paulo AC (1999). Diphtheria, pertussis, and measles in Portugal before and after mass vaccination: a time series analysis. Eur J Epidemiol.

[7] Metcalf CJE, Bjørnstad ON, Grenfell BT, Andreasen V (2009). Seasonality and comparative dynamics of six childhood infections in pre-vaccination Copenhagen. Proc Biol Sci.

[8] Anderson RM, Grenfell BT, May RM (1984). Oscillatory fluctuations in the incidence of infectious disease and the impact of vaccination: time series analysis. J Hyg (Lond).

[9] Cherry JD (2013). Pertussis: challenges today and for the future. PLoS Pathog.

[10] Rohani P, Zhong X, King AA (2010). Contact network structure explains the changing epidemiology of pertussis. Science.

[11] Robert Koch Institut. Infektionsepidemiologisches Jahrbuch meldepflichtiger Krankheiten für 2016: Jahresstatistik nach Bundesland; 2017 [cited 2019 Nov 25]. Available from: URL: https://www.rki.de/DE/Content/Infekt/Jahrbuch/Jahresstatistik_2016.pdf?__blob=publicationFile.

[12] Robert Koch Institut. Infektionsepidemiologisches Jahrbuch meldepflichtiger Krankheiten für 2017: Jahresstatistik nach Bundesland; 2018 [cited 2018 Nov 29]. Available from: URL: https://www.rki.de/DE/Content/Infekt/Jahrbuch/Jahresstatistik_2017.pdf?__blob=publicationFile.

[13] Bhatti MM, Rucinski SL, Schwab JJ, Cole NC, Gebrehiwot SA, Patel R (2017). Eight-year review of Bordetella pertussis testing reveals seasonal pattern in the United States. J Pediatric Infect Dis Soc.

[14] European Centre for Disease Prevention and Control. Annual Epidemiolocal Report for 2016. Pertussis. Stockholm: European Centre for Disease Prevention and Control; 2018. Available from: URL: https://www.ecdc.europa.eu/en/publications-data/pertussis-annual-epidemiological-report-2016. Accessed 28 Mar 2019.

[15] Zeng Q, Li D, Huang G, Xia J, Wang X, Zhang Y (2016). Time series analysis of temporal trends in the pertussis incidence in mainland China from 2005 to 2016. Sci Rep.

[16] Kaczmarek MC, Ware RS, Nimmo GR, Robson JMB, Lambert SB (2016). Pertussis seasonality evident in polymerase chain reaction and serological testing data, Queensland, Australia. J Pediatric Infect Dis Soc.

[17] Dalby T, Andersen PH, Hoffmann S. Epidemiology of pertussis in Denmark, 1995 to 2013. Euro Surveill. 2016;21(36):30334. 10.2807/1560-7917.ES.2016.21.36.30334. 10.2807/1560-7917.ES.2016.21.36.30334 PMC5048713 27632433

[18] Veneable D, Miro-Quesada G, Calley J, Monson E, He L (2007). High-throughput and quantitative detection of residual NS0 and CHO host cell genomic DNA. BioProcess Int.

[19] Roorda L, Buitenwerf J, Ossewaarde JM, van der Zee A (2011). A real-time PCR assay with improved specificity for detection and discrimination of all clinically relevant Bordetella species by the presence and distribution of three insertion sequence elements. BMC Res Notes.

[20] Tatti KM, Sparks KN, Boney KO, Tondella ML (2011). Novel multitarget real-time PCR assay for rapid detection of Bordetella species in clinical specimens. J Clin Microbiol.

[21] Pittet LF, Emonet S, François P, Bonetti E-J, Schrenzel J, Hug M (2014). Diagnosis of whooping cough in Switzerland: differentiating Bordetella pertussis from Bordetella holmesii by polymerase chain reaction. PLoS One.

[22] RStudio: Integrated Development Environment for R. Boston, MA: RStudio, Inc.; 2018. Available from: URL: www.rstudio.com. Accessed 12 Sept 2019.

[23] de Greeff SC, Dekkers ALM, Teunis P, Rahamat-Langendoen JC, Mooi FR, de Melker HE (2009). Seasonal patterns in time series of pertussis. Epidemiol Infect.

[24] Miyashita N, Akaike H, Teranishi H, Kawai Y, Ouchi K, Kato T (2013). Diagnostic value of symptoms and laboratory data for pertussis in adolescent and adult patients. BMC Infect Dis.

[25] Robert Koch-Institut. Falldefinitionen des Robert Koch-Instituts zur Übermittlung von Erkrankungs-oder Todesfällenund Nachweisen von Krankheitserregern: Robert Koch-Institut; 2019 [Cited 2019 Dec 16]. Available from: URL: https://www.rki.de/DE/Content/Infekt/IfSG/Falldefinition/Downloads/Falldefinitionen_des_RKI_2019.pdf?__blob=publicationFile. 10.1007/s00103-016-2355-2 27221548

[26] Robert Koch-Institut. Infektionsepidemiologisches Jahrbuch meldepflichtiger Krankheiten für 2017: 6.29 Keuchhusten [Datenqualität]; 2018 [cited 2019 Nov 25]. Available from: URL: https://www.rki.de/DE/Content/Infekt/Jahrbuch/Jahrbuch_2017.pdf?__blob=publicationFile.

[27] King AA, Domenech de Cellès M, Magpantay FMG, Rohani P. Pertussis immunity and the epidemiological impact of adult transmission. In: Rohani P, Scarpino SV, editors. Pertussis: Epidemiology, immunology, and evolution. First edition. Oxford, United Kingdom: Oxford University Press; 2019. p. 225–40 (Ecology and evolution of infectious diseases series).

[28] Domenech de Cellès M, Magpantay FMG, King AA, Rohani P. The impact of past vaccination coverage and immunity on pertussis resurgence. Sci Transl Med. 2018;10(434):eaaj1748. 10.1126/scitranslmed.aaj1748. 10.1126/scitranslmed.aaj1748 PMC6063734 29593103

[29] Skowronski DM, de Serres G, MacDonald D, Wu W, Shaw C, Macnabb J (2002). The changing age and seasonal profile of pertussis in Canada. J Infect Dis.

